# Hyperpolarized ^13^C spectroscopic imaging using single‐shot 3D sequences with unpaired adiabatic refocusing pulses

**DOI:** 10.1002/nbm.4004

**Published:** 2018-09-10

**Authors:** Jiazheng Wang, Richard L. Hesketh, Alan J. Wright, Kevin M. Brindle

**Affiliations:** ^1^ Cancer Research UK Cambridge Institute University of Cambridge Li Ka Shing Centre Cambridge UK; ^2^ Department of Biochemistry University of Cambridge Cambridge UK

**Keywords:** hyperpolarization, imaging, lactate, pyruvate, tumors

## Abstract

Hyperpolarized MRI with ^13^C‐labeled metabolites has enabled metabolic imaging of tumors in vivo. The heterogeneous nature of tumors and the limited lifetime of the hyperpolarization require high resolution, both temporally and spatially. We describe two sequences that make more efficient use of the ^13^C polarization than previously described single‐shot 3D sequences. With these sequences, the target metabolite resonances were excited using spectral‐spatial pulses and the data acquired using spiral readouts from a series of echoes created using a fast‐spin‐echo sequence employing adiabatic 180° pulses. The third dimension was encoded with blipped gradients applied in an interleaved order to the echo train. Adiabatic inversion pulses applied in the absence of slice selection gradients allowed acquisition of signal from odd echoes, formed by unpaired adiabatic pulses, as well as from even echoes. The sequences were tested on tumor‐bearing mice following intravenous injection of hyperpolarized [1‐^13^C]pyruvate. [1‐^13^C] pyruvate and [1‐^13^C] lactate images were acquired in vivo with a 4 × 4 × 2 cm^3^ field of view and a 32 × 32 × 16 matrix, leading to a nominal resolution of 1.25 × 1.25 × 1.25 mm^3^ and an effective resolution of 1.25 × 1.25 × 4.5 mm^3^ when the *z*‐direction point spread function was taken into account. The acquisition of signal from more echoes also allowed for an improvement in the signal‐to‐noise ratio for resonances with longer *T*_2_ relaxation times. The pulse sequences described here produced hyperpolarized ^13^C images with improved resolution and signal‐to‐noise ratio when compared with similar sequences described previously.

**Abbreviations**
CPMG: Carr‐Purcell‐Meiboom‐Gill
CSI: chemical shift imaging
DSE: dual spin echo
EPI: echo planar imaging
EPSI: echo‐planar‐based spectroscopic imaging
FOV: field of view
FSE: fast spin echo
FWHM: full width at half maximum
HS: hyperbolic secant
MRSI: magnetic resonance spectroscopic imaging
PSF: point spread function
SpSp: spatial‐spectral excitation

## 1 INTRODUCTION

Magnetic resonance spectroscopic imaging (MRSI) with hyperpolarized ^13^C‐labelled metabolites has provided a new way to study metabolism in vivo,^1^, ^2^, ^3^ which has translated to the clinic.^4^, ^5^ Pyruvate has been the most intensively investigated metabolite because of its central role in metabolism, the ease with which it can be hyperpolarized and because its membrane transport and metabolism are fast when compared with the lifetime of the polarization.^6^ Imaging exchange of hyperpolarized ^13^C label between injected hyperpolarized [1‐^13^C]pyruvate and endogenous tumor lactate can be used to assess treatment response,^7^ detect occult disease,^4^ and monitor disease progression.^8^, ^9^ There are four main challenges in pulse sequence design for hyperpolarized ^13^C MRI^3^: (i) the number of pulses should be kept to a minimum, as each excitation depletes polarization; (ii) spatial resolution should be as high as possible; (iii) image acquisition should be fast to cope with the rapidly decaying polarization; and (iv) spectral resolution should be sufficient to resolve signals from different metabolites in the resulting images. Many imaging pulse sequences have been developed to meet these demands. Cunningham et al. proposed an echo‐planar‐based spectroscopic imaging (EPSI) technique for mapping multiple metabolites^10^ and Larson and colleagues combined this with multiband spectral‐spatial excitations in order to optimize use of the polarization in individual metabolites.^11^ Compressed sensing has been used to enhance imaging efficiency,^12^ exploiting the sparse nature of the hyperpolarized signal, both spatially and spectrally. This led subsequently to the development of a highly accelerated EPSI technique that exploited both multiband spectral‐spatial excitation (SpSp) pulses and compressed sensing.^13^ Others have used single‐band SpSp pulses to image a single metabolite at a time, for example with 2D and 3D echo planar imaging (EPI),^14^, ^15^ which can be accelerated with compressed sensing.^16^ The EPI trajectory can also be designed to enable separation of metabolite resonances in the resulting images.^17^ Spiral chemical shift imaging (CSI) has been studied extensively,^18^, ^19^, ^20^ while traditional CSI with compressed sensing has also shown promise for dynamic measurements.^21^ 2D spatial and 1D spectral information can also be acquired after a single excitation by exploiting spatiotemporal encoding.^22^ We recently proposed a single‐shot 3D sequence that makes efficient use of the transverse magnetization after each excitation.^23^ The sequence exploits the *B*_1_ insensitivity of adiabatic pulses, following a dual‐spin‐echo (DSE) scheme that is widely used in other hyperpolarized MRI applications.^10^, ^11^, ^12^, ^17^, ^24^, ^25^ The adiabatic pulses in the DSE scheme result in better refocusing and hence less signal loss due to dephasing, but the pulse sequence is inefficient in its use of spin hyperpolarization, since signal is acquired from only half of the spin echoes. Here we propose an enhanced version of this single‐shot 3D sequence that uses unpaired adiabatic inversion pulses for refocusing and doubles the number of encoding steps in the *z* direction with the same number of pulses. We also describe an alternative design that can significantly enhance the signal‐to‐noise ratio (SNR), at the expense of a shorter imaging time window.

## 2 METHODS

### 2.1 Tumor imaging

Tumors were obtained by subcutaneous implantation of EL4 murine lymphoma cells, as described in Reference 23. Mice were fasted for 6–8 h before imaging.^26^ Images were acquired at 7 T and the [1‐^13^C] pyruvate was hyperpolarized as described previously.^23^ Experiments were performed in compliance with a project license issued under the Animals (Scientific Procedures) Act of 1986. Protocols were approved by the Cancer Research UK, Cambridge Institute Animal Welfare and Ethical Review Body.

### 2.2 MRI scanner

Experiments were performed at 7 T (Agilent, Palo Alto, CA, USA), with a 42 mm diameter bird‐cage volume coil for ^1^H transmission and reception, and a similar volume coil for ^13^C transmission and a 20 mm diameter surface coil for ^13^C detection (Rapid Biomedical, Rimpar, Germany).

### 2.3 Pulse sequence

**Figure 1:**
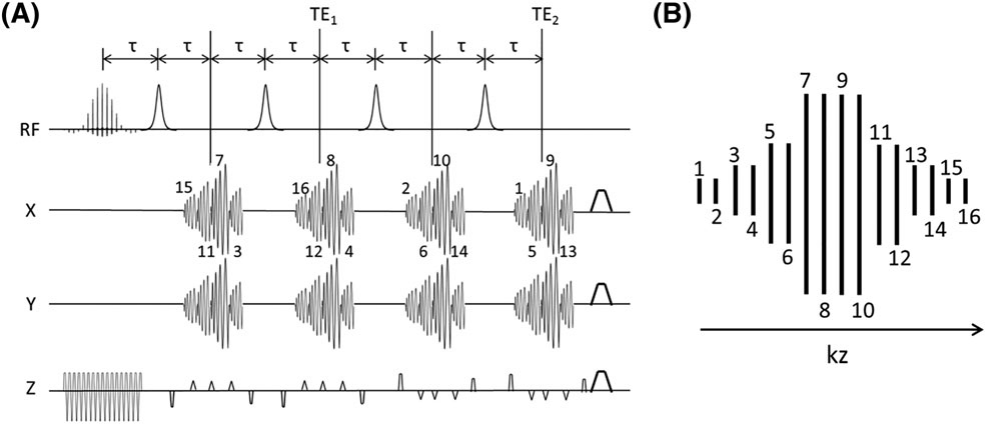
The proposed pulse sequence and its *k*‐space trajectory in the *k*_*z*_ direction. A, FSE‐I pulse sequence, which starts with an SpSp pulse and a train of four adiabatic refocusing pulses. A stack of four spiral acquisitions is acquired after each refocusing pulse, and each spiral is phase encoded in the *z* direction by blipped gradients on the *z* axis. For the FSE‐II sequence four extra stacks of spiral acquisitions are acquired with the same refocusing pulses and phase encodings in the *z* direction as in the first four stacks. The two signal blocks are then averaged in order to improve SNR. The FSE‐II sequence hence shares the same *k*‐space trajectories as the FSE‐I pulse sequence. B, *k*‐space trajectory in the *k*_*z*_ direction. Each spiral index in the sequence in A corresponds to a *k*_*z*_ position in B

**Figure 2:**
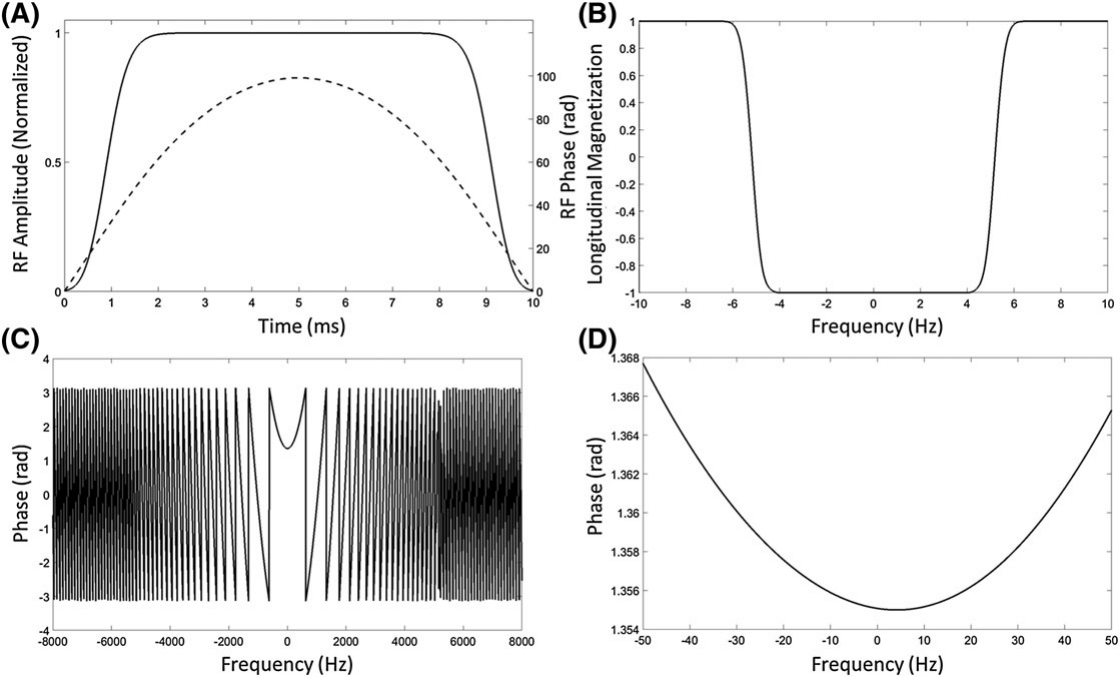
Adiabatic pulse design. A, *B*_1_ (solid line) and phase (dashed line) waveforms. B, Simulation of the inversion profile. The pulse was designed to have a bandwidth of 10 kHz and to achieve more than 99.9% inversion over a frequency band of ±4 kHz at a *B*_1_ field strength between 100 and 6400 μT. C, The quadratic phase induced in transverse magnetization when the pulse is applied to magnetization lying on the *y* axis. The same quadratic phase could be observed across the whole slice when a slice selection gradient was applied. D, The quadratic phase variation is small within a frequency band of ±50 Hz of the frequency offset of the pulse

Target metabolite resonances were excited using a 10.016 ms SpSp pulse^23^ with a bandwidth of 350 Hz and 1645 Hz (center to center) between replicate excitation bands. A 90° pulse required a *B*_1_ field of 131.11 μT and used a fly‐back trajectory to reduce vulnerability to eddy currents, with maximum gradient strengths of 0.19 and 0.2993 T/m at positive and negative polarities, giving a 1.2 cm minimum slab thickness. A fast‐spin‐echo (FSE) train, with four refocusing pulses, followed the excitation pulse, with a stack of four spiral acquisitions during each spin echo (Figure 1A). Removal of slice selection gradients from the refocusing pulses used previously in a similar sequence^23^ allowed signal acquisition from both even and odd echoes. Previously spirals were not acquired from odd echoes because a fully refocused spin echo, in the presence of slice selection gradients, requires a pair of hyperbolic secant (HS) adiabatic pulses,^27^ otherwise a quadratic phase is left across the swept frequency range, as illustrated in Figure 2C. This non‐linear phase generated by an unpaired HS pulse results in signal loss as each *k*‐space point is the summation of signals from a range of frequencies. When this range of frequencies is large, as is the case when a slice selection gradient is used, then signal loss is considerable. In the absence of a slice selection gradient the frequency range over which there is phase variation depends only on local *B*_0_ field variation. In a reasonably well‐shimmed *B*_0_ field this frequency range is much smaller (of the order of ±50 Hz as compared with ±5 kHz when a slice selection gradient is present) and moreover shows a smooth variation, leading to only mild signal loss (Figure 2D). Each spiral is encoded in the *xy* plane and each stack of spirals is phase encoded in the *z* direction with blipped gradients such that a 3D *k*‐space is acquired. The *z*‐dimension information is phase encoded in an interleaved manner and the *k*_*z*_ index of each spiral (Figure 1B) in each stack is given in the following:

Stack 1—15, 11, 7, 3
Stack 2—16, 12, 8, 4
Stack 3—2, 6, 10, 14
Stack 4—1, 5, 9, 13.

The phase‐encoding gradients on the *z* axis are self‐refocusing in each acquisition interval in order to comply with the Carr‐Purcell‐Meiboom‐Gill (CPMG) condition. In each acquisition interval the spiral dimensions in the *k*_*xy*_ plane are 4 × 4, 16 × 16, 32 × 32, 8 × 8, in the order displayed in Figure 1A, and the spiral dimensions for the 3D *k*‐space are thus (Figure 1B, from *k*_*z*1_ to *k*_*z*16_) 4 × 4, 4 × 4, 8 × 8, 8 × 8, 16 × 16, 16 × 16, 32 × 32, 32 × 32, 32 × 32, 32 × 32, 16 × 16, 16 × 16, 8 × 8, 8 × 8, 4 × 4, and 4 × 4. The durations for the 4 × 4, 8 × 8, 16 × 16, and 32 × 32 spirals are 0.552, 1.216, 3.120, and 8.500 ms respectively. Each spiral encodes a field of view (FOV) of 4 × 4 cm^2^ in the *xy* plane. This sequence is referred to as FSE‐I hereafter. An alternative sequence (hereafter referred to as FSE‐II) can be constructed such that the same *z*‐dimension phase encoding as in Figure 1A is repeated in additional stacks 5 to 8 and the sampled points are averaged with the datasets acquired through stacks 1 to 4 (see Figure S1). A limitation of this trajectory design is that the less densely sampled regions in the corners of 3D *k*‐space lead to a widened point spread function (PSF) in the *z* direction, which was investigated with the PSF simulations described below. A spherical or cylindrical 3D *k*‐space would improve the *z*‐direction PSF and enhance resolution in the *z* direction.

Imperfect refocusing pulses can dramatically deplete the polarization, especially within their transition bands. Therefore adiabatic inversion pulses with hyperbolic‐secant modulation (HS*n*, *n* = 8)^28^ were used for refocusing, where each had a duration of 10 ms (Figure 2A) and a bandwidth of 10 000 Hz (Figure 2B shows the effect of the pulse on the longitudinal magnetization at a *B*_1_ field strength of 100 μT). The pulses were designed to achieve perfect inversion (>99.9% of the longitudinal magnetization inverted) when the *B*_1_ field was between 100 and 6400 μT. The large time‐bandwidth product also ensured insensitivity to *B*_0_ inhomogeneity and that there was only a small and smooth phase variation in the frequency response so that signal loss was minimized. The phase response of the pulse, when applied along the *y* axis, to the *y* magnetization is shown in Figure 2C. The central part of this phase response is expanded in Figure 2D.

To preserve the CPMG condition, the delay *τ* in Figure 1A was 15.284 ms, which was determined by the durations of the excitation and inversion pulses and the spirals. Since there are two spirals (eighth and ninth) acquired at the center of the *k*_*z*_ direction, the resulting contrast is contributed by two different TE times (61.136 and 122.272 ms), which, in the FSE‐II sequence, are averaged with another two echoes at 183.408 and 244.544 ms.

### 2.4 Simulations

The PSF of the acquisition trajectory was simulated for the proposed sequences, where a constant *k*‐space was sampled by the proposed *k*‐space trajectory and 3D Fourier transformed into the image domain.^29^ This was then compared with the PSF obtained using the forerunner of these sequences, the DSE sequence.^23^ Despite additional signal obtained from the later echoes, the FSE‐II sequence has the same *k*‐space trajectory and hence the same PSF as the FSE‐I sequence when relaxation is ignored.

### 2.5 Pulse calibration

The adiabatic pulse was calibrated on a cylindrical phantom (7 mm inner diameter) filled with 5 M [1‐^13^C]lactate at thermal equilibrium. A pulse‐acquire sequence without any gradients was used, where 2048 data points were acquired at 4 μs intervals immediately after the pulse. The sequence swept a range of frequencies (from 0 Hz to 4500 Hz offset, with 300 Hz step size) and *B*_1_ field strengths (from 40 to 470 μT). The inversion pulses were also tested on a cylindrical phantom of the same size injected with hyperpolarized [1‐^13^C] pyruvate and imaged using both the FSE‐I and FSE‐II sequences. All the gradients were turned off except for the slice selection gradient for the SpSp excitation pulse. To mimic the RF exposure in experiments in vivo, the SpSp pulse was targeted alternately to the [1‐^13^C] pyruvate and [1‐^13^C] lactate resonance frequencies, with flip angles of 7° and 45° respectively, a slice thickness of 20 mm, and a TR of 1 s (hence 2 s TR at each frequency). The whole acquisition lasted for 90 s.

### 2.6 Phantom imaging

The original pulse sequence (DSE sequence^23^) and the FSE‐I sequence were tested on the cylindrical phantom containing thermally polarized [1‐^13^C] lactate. One end of the phantom was positioned at the iso‐center (also the center of the FOV in the *z* direction) to examine the definition of the resulting images. The FOVs for both sequences were 4 × 4 × 2 cm^3^ in *x*, *y*, and *z* dimensions, with eight phase‐encoding steps in the *z* direction in the original DSE sequence and 16 steps in the FSE‐I sequence. Identical spiral designs were used for the DSE sequence, which were the same as those described in Reference 23, except for the optimized refocusing lobe at the end of each spiral. The flip angle was 90° for both sequences, exciting a slab of 12 mm. The excited slab was thinner than the *z*‐direction FOV to avoid wrap‐around artifacts from both the transition band of the excitation pulse and the side lobes of the PSF in the *z* direction. The phantom was then repositioned with the center of the phantom at the iso‐center in order to compare the SNR between the three sequences. To investigate signal loss caused by the quadratic phase imparted by unpaired adiabatic pulses, signal was acquired using the DSE and FSE‐I sequences with the encoding gradients turned off on all three axes. The PSFs of the FSE‐I and FSE‐II sequences were simulated again by adding a weighting factor for signal decay to each *k*_*z*_ plane using the measured signal loss due to unpaired adiabatic pulses, the [1‐^13^C] lactate *T*_2_ value measured in the phantom (1 s), and a *T*_2_ value (300 ms) estimated in vivo at 7 T.^25^ The simulation was then repeated using a *T*_2_* decay of 12 ms measured at 7 T.^30^

FSE proton images were acquired as 16 slices to cover the same volume as the ^13^C images, with a 4 × 4 cm^2^ FOV, 1.25 mm slice thickness, and 256 × 256 matrix size.

### 2.7 Imaging of hyperpolarized [1‐^13^C] pyruvate and [1‐^13^C] lactate in vivo

Acquisition of [1‐^13^C] pyruvate images (FSE‐I was used with two mice and FSE‐II with three mice) started 2 s after tail vein injection of hyperpolarized [1‐^13^C] pyruvate. Five [1‐^13^C] pyruvate images were acquired at a 2 s frame rate before the first [1‐^13^C] lactate image was acquired, 1 s after the fifth [1‐^13^C] pyruvate image. [1‐^13^C] pyruvate and [1‐^13^C] lactate images were acquired in alternating order thereafter, hence there was a temporal resolution of 2 s/frame for each metabolite. The flip angles on [1‐^13^C] pyruvate and [1‐^13^C] lactate were 7° and 45° respectively. This helps to preserve the polarization for [1‐^13^C] pyruvate and enhances the image SNR for [1‐^13^C] lactate. The eighth pair of acquisitions was used as a reference scan, where the *z*‐axis gradients were turned off except for the slice selection gradient and the spoiler. The FOVs in all three dimensions were 4 × 4 × 2 cm^3^. The excitation bandwidth was 12 mm at half maximum to avoid wrap‐around artifacts in the *z* direction. The total acquisition window was 90 s. The water proton frequency was used to estimate the [1‐^13^C] pyruvate and [1‐^13^C] lactate resonance frequencies. *T*_2_‐weighted proton images (FSE, 128 × 128 matrix, 16 slices, 1.25 mm slice thickness) were acquired for positional reference.

### 2.8 Image reconstruction

^13^C MRI images were reconstructed in MATLAB (MathWorks, Natick, MA, USA), as described in Reference 23.

## 3 RESULTS

The adiabatic pulses achieved almost complete inversion at ±3500 Hz when the *B*_1_ field was greater than 120 μT, matching their expected performance (Supplementary Figure S2A). Signals acquired from a phantom injected with hyperpolarized [1‐^13^C] pyruvate, using both the FSE‐I and FSE‐II sequences, are shown in Supplementary Figure S2B. Fitting the time course of signal decay gave a *T*_1_ of 50 s for the FSE‐I sequence and 48 s for the FSE‐II sequence, indicating that additional polarization depletion, incurred by doubling the number of inversion pulses in the FSE‐II sequence, was negligible.

**Figure 3:**
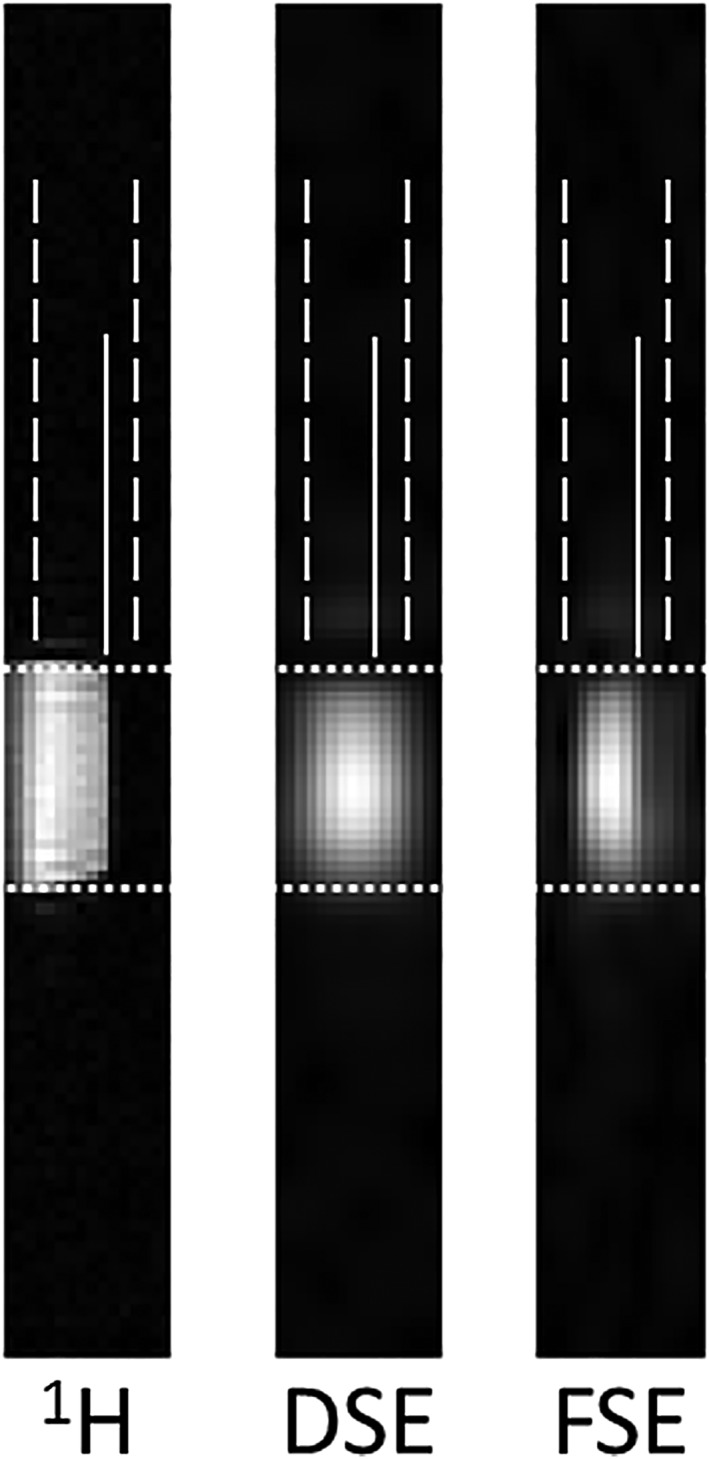
The proposed FSE sequences enhance resolution in the *z* direction from a phantom (7 mm inner diameter) filled with thermally polarized [1‐^13^C] lactate. The dashed lines indicate the position of the excited slab and the solid lines indicate the edge of the phantom in the *z* direction. The phantom edges in the other dimension are marked by dotted lines. A high‐resolution proton image is shown for positional reference

The simulated PSFs in the *xy* plane (Supplementary Figure S3A) for the DSE and FSE sequences were slightly different because of differences in the 3D *k*‐space trajectories. However the FSE sequences halved the bandwidth in the *z* direction when compared with the DSE sequence (Supplementary Figures S3A and S3B), which doubled the *z*‐direction resolution in the resulting image. This improved resolution on the *z* axis was confirmed experimentally with images acquired from a phantom containing 5 M [1‐^13^C] lactate at thermal equilibrium (Figure 3). There was almost no signal outside the excited slab in the FSE image, whereas signal could be observed in the DSE image. However, the full width at half maximum (FWHM) of the PSF, even for the FSE sequence, is about 4 times the nominal *z*‐direction resolution (4.5 mm versus 1.25 mm). This is because the corners of 3D *k*‐space were under‐sampled with this spiral trajectory.

**Figure 4:**
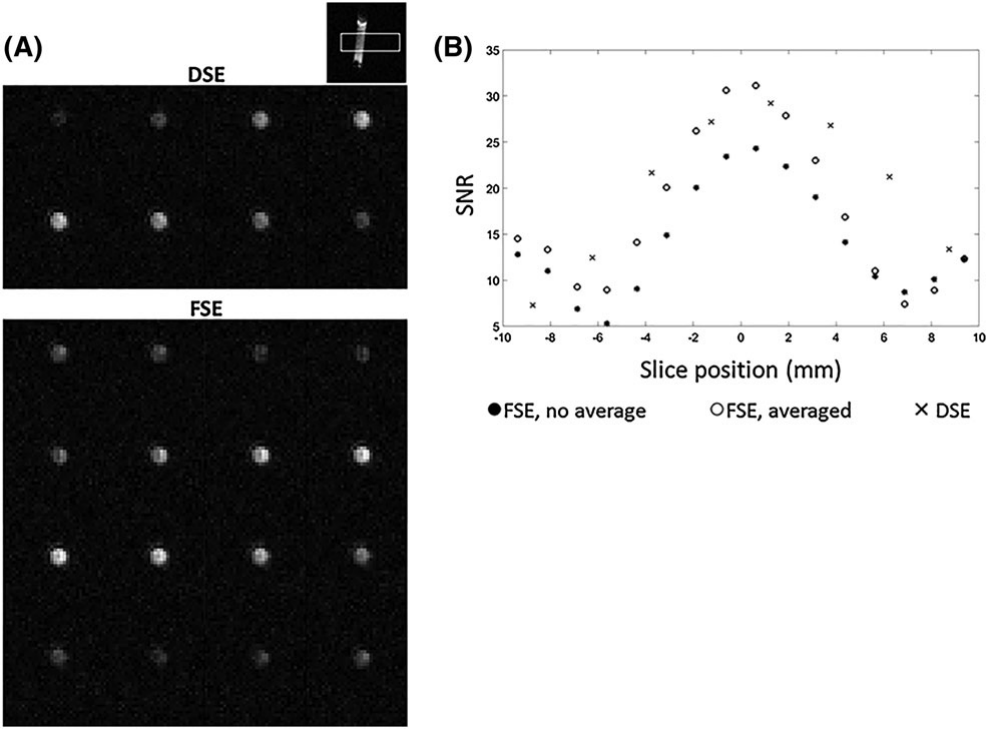
A, Images acquired with the DSE and FSE‐II pulse sequences from a cylindrical phantom containing 5 M [1‐^13^C] lactate at thermal equilibrium. B, Signal from the first four echoes in the FSE‐II pulse sequence (equivalent to the FSE‐I pulse sequence) and signal obtained by averaging the signal from the first four echoes with the last four echoes in the FSE‐II pulse sequence

Axial images acquired using the DSE and FSE‐II sequences, when the center of the phantom was at magnet center (Figure 4A), showed that the SNRs of the two central *z* slices acquired using the FSE‐I sequence were 0.86 and 0.83 times the SNRs acquired using the DSE sequence, reflecting the improved resolution, while the SNRs in slices acquired using the FSE‐II sequence were 1.12 and 1.07 times the DSE SNRs (Figure 4B). Expressing the SNR per unit volume, the FSE‐I SNRs were 1.72 and 1.66 times the DSE SNRs and the FSE‐II SNRs were 2.25 and 2.13 times greater. The SNR curves in Figure 4B show the slab profile for each sequence, which was in agreement with convolution of the slice response of the excitation pulse (Figure 1C of Reference 23) with the PSFs in the *z* direction for each sequence.

**Figure 5:**
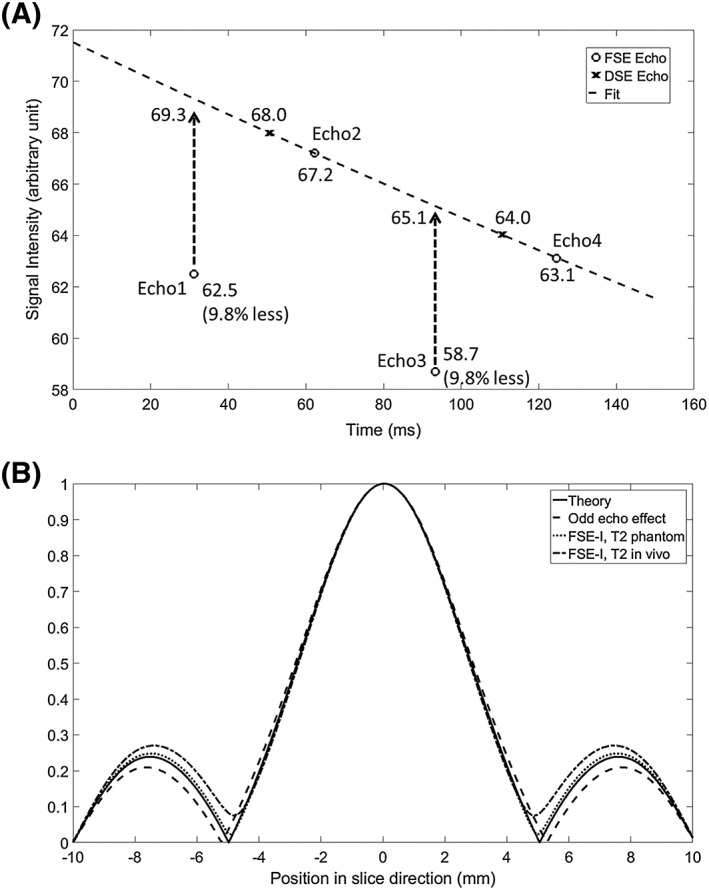
A, Signal loss in echoes formed with unpaired adiabatic refocusing pulses. DSE and FSE‐I sequences were used to acquire signal from the lactate phantom shown in Figure 4, with all encoding gradients turned off. The second and fourth echoes acquired with FSE‐I were fitted to give a curve that shows *T*_2_ relaxation. The fitted curve matched well with the echoes acquired using the DSE pulse sequence. The first and third echoes in the FSE‐I sequence, which were formed by unpaired adiabatic pulses, were 9.8% smaller than the echoes that would have been formed had there been no adiabatic phase twist (as indicated by the curve showing signal loss due to *T*_2_ relaxation). B, Simulated PSFs, showing the theoretical case (solid line), the effects of signal loss at odd echoes (dashed line), and the effects of *T*_2_ relaxation, as measured in the phantom (dotted line) and in vivo (dash‐dotted line)

Phantom data were also acquired with encoding gradients on all three axes turned off. The two acquired echoes in the DSE sequence and the four acquired echoes in the FSE‐I sequence are shown in Figure 5A. The *T*_2_ decay curve was obtained by fitting to the amplitude of the even echoes in the FSE‐I sequence, as these were formed with paired adiabatic pulses and hence free of quadratic phase twist and thus loss of signal. Signals from the odd echoes in the FSE sequence were approximately 10% lower than expected from signal decay due to *T*_2_ relaxation. This decrease was greater than that expected from the non‐linear phase shift imparted by the adiabatic pulses (Figure 5A) and imperfections in local *B*_0_ and *B*_1_ fields, and could be due to mis‐setting of the frequency of the RF pulse and small shifts in the timing of the echoes in the presence of the adiabatic pulses. As shown in Figure 5B, signal loss due to unpaired adiabatic pulses increases slightly the width of the main lobe of the PSF by 2.3% while reducing the amplitude and bandwidth of the side lobes by 12.3% and 5.5%, respectively. However, these effects disappear when *T*_2_ decay is taken into account for both FSE‐I (Figure 5B) and FSE‐II sequences (not shown). When *T*_2_* decay was considered, the main lobe of the PSF was increased by 2.9% and the side lobe by 3.2%, compared with the *T*_2_ decay only case, for both FSE‐I and FSE‐II sequences. The echoes from the central *k*‐space in the FSE sequence were smaller in amplitude than their counterparts in the DSE sequence because the timing of the pulse train in the FSE sequence is different from that in the DSE sequence. In the DSE sequence, the minimum interval between the excitation pulse and the first refocusing pulse is determined solely by the pulse durations, while in the FSE sequence this interval also depends on the durations of the first and second spirals.

**Figure 6:**
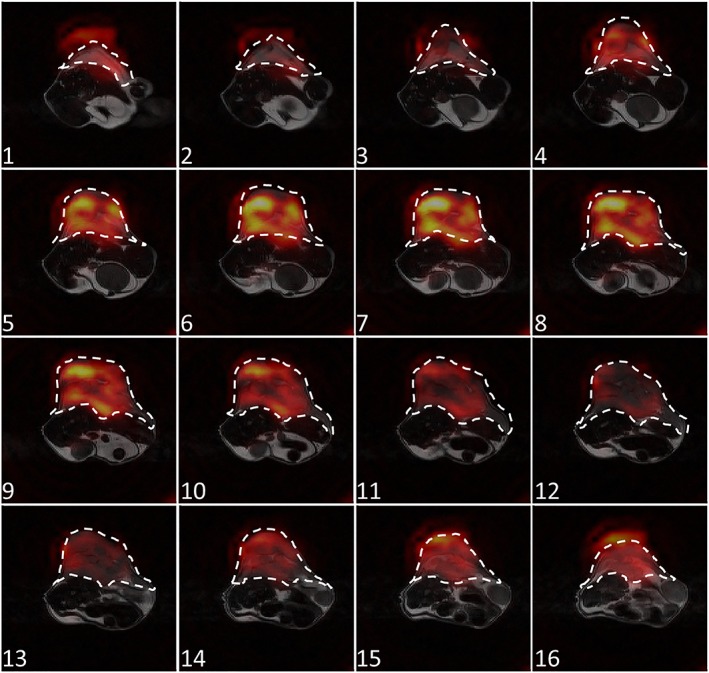
Representative [1‐^13^C] lactate images acquired in vivo using the FSE‐II pulse sequence. All 16 *z*‐axis slices from a single frame (10 s from the start of acquisition) are shown. The slices are indexed in the tail to head direction. Unwanted signals were observed in slices at both ends in the *z* direction, due to ripples in the *z*‐direction PSF. The location of the tumor is indicated by a dashed white line. Corresponding [1‐^13^C] pyruvate images can be found in Supplementary Figure S5

**Figure 7:**
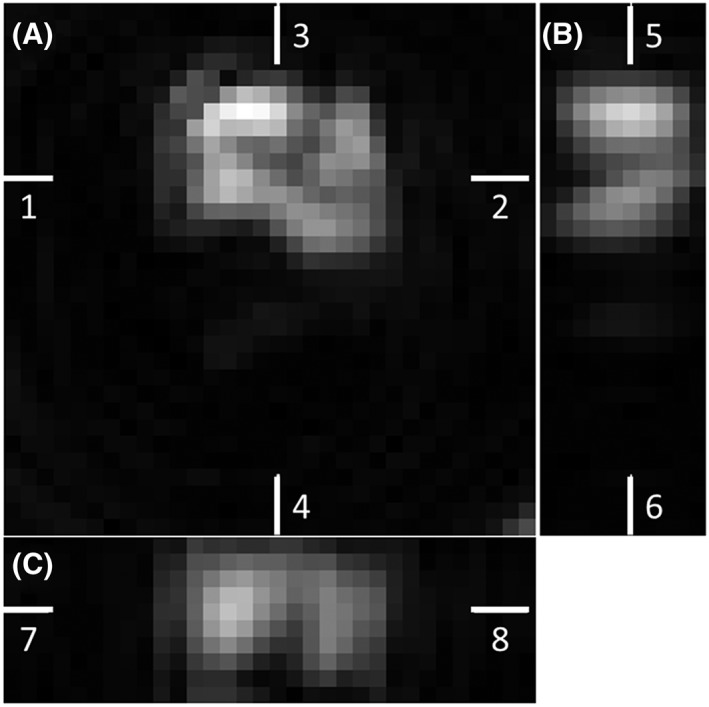
Sagittal and coronal views reconstructed from the central 10 slices from the [1‐^13^C] lactate images shown in Figure 6. The ninth slice is shown as a representative axial plane image. The white bars indicate the positions of each displayed image in the 3D volume. A, Axial plane, position indicated by Bars 5 and 6 in B and 7 and 8 in C. B, Sagittal plane, position indictated by Bars 3 and 4 in A. C, Coronal plane, position indicated by Bars 1 and 2 in A. Heterogeneity could be observed in both the *xy* plane and the *z* direction

**Figure 8:**
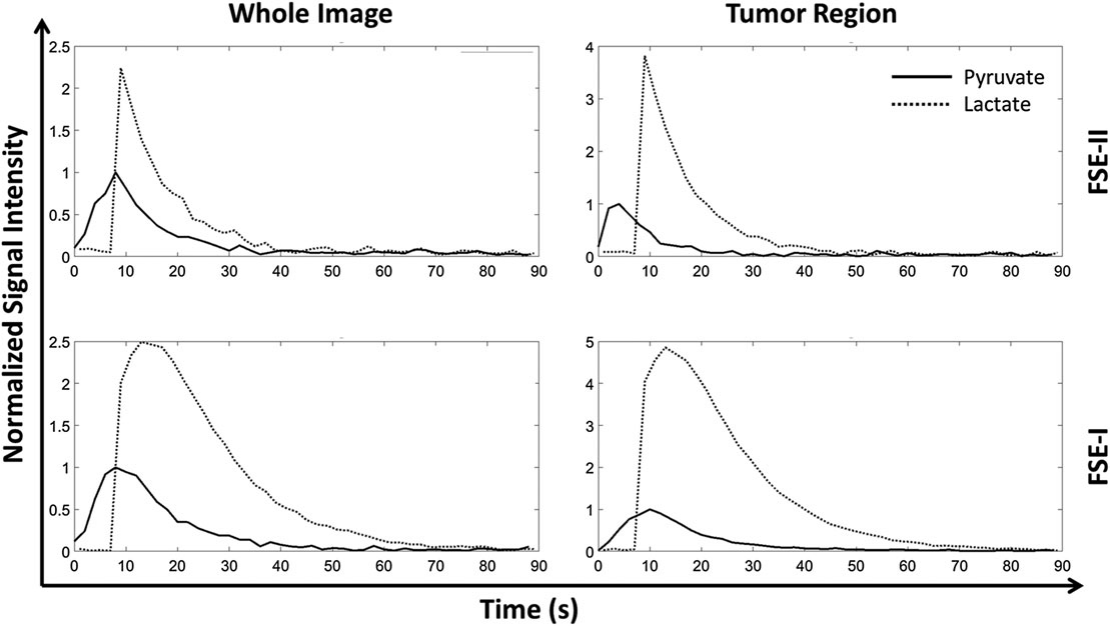
Representative pyruvate and lactate signal intensities in images acquired with the FSE‐I and FSE‐II pulse sequences from tumor‐bearing mice injected with hyperpolarized [1‐^13^C] pyruvate. Signal intensities in the whole image and from the tumor region are plotted as a function of time after pyruvate injection. Each dataset was normalized to the peak value in the [1‐^13^C] pyruvate time course

Representative [1‐^13^C] lactate images acquired using the FSE‐II sequence following i.v. injection of hyperpolarized [1‐^13^C] pyruvate in a tumor‐bearing mouse are shown in Figure 6. The corresponding [1‐^13^C] pyruvate images are displayed in Supplementary Figure S5. [1‐^13^C] lactate images in all three planes (axial, sagittal, and coronal) show considerable heterogeneity (Figure 7). Only the central 10 slices (in the *z* direction) are displayed in the sagittal and coronal images, as the excitation slab was 12 mm. Representative time courses of signal intensities from the whole image and from the tumor region, acquired with the FSE‐I and FSE‐II sequences from the central 10 slices, are shown in Figure 8. The SNR enhancements for lactate in the FSE‐II sequence, obtained by averaging signal from the earlier echoes with the later echoes, for the central four slices in Frames 5 to 9, which were the frames with the highest SNR, were 32 ± 8%, 19 ± 2%, and 1.8 ± 13.7% for the three animals imaged. The time course data from two mice using the FSE‐I sequence and all three mice using the FSE‐II sequence are shown in Supplementary Figure S6.

## 4 DISCUSSION

While the previous DSE sequence provided high temporal and spatial resolution,^23^ the FSE sequences described here doubled the spatial resolution in the *z* direction with no sacrifice of temporal resolution. Although the FSE‐II sequence doubled the sequence length from 125 ms to 247 ms, in practice the frame rate is rarely set to below 250 ms/frame, as this would lead to fast depletion of the polarization.

Although the PSF of the FSE sequences is much sharper in the *z* direction, it has a pair of side lobes toward the ends of the *z*‐direction FOV, which may result in image artifacts. Such artifacts can be observed in Figure 6. The slices at both ends of the *z*‐direction FOV (20 mm) should show no signal due to the limited thickness of the excitation slab (12 mm). These artifacts are less obvious in images acquired with the DSE sequence, as they appear as image blur in the *z* direction. A window function could be applied to the *k*‐space in the *k*_*z*_ direction to reduce these artifacts, but this will render a PSF more similar to that of the DSE sequence and compromise the enhancement in image definition. Alternatively, the end slices could be discarded from the final set of images. Simulations of the PSF in the phantom experiments showed that modulation, caused by both *T*_2_ decay and signal loss in odd echoes, was negligible (Figure 5B). Faster *T*_2_ decay in vivo may result in greater modulation of the PSF, which is a common concern for FSE sequences, while the effects of signal acquisition from odd echoes should be relatively minor as the *B*_0_ field is usually well shimmed on pre‐clinical systems. In further simulations, the widths of the main lobes of the PSFs of the FSE sequences at a *T*_2_ of 50 ms were half way between the widths in the FSE and DSE sequences when relaxation was ignored, demonstrating a resolution benefit for the FSE over the DSE sequence, even for metabolites with very short *T*_2_ times. *T*_2_* decay during each acquisition interval can cause signal variations between the four spirals in each interval, which in general suppresses signals from the edge spirals in the 3D *k*‐space and hence blurring in the *z* direction. However, the short durations of each spiral reduce this effect considerably. Moreover, simulations with *T*_2_* = 12 ms, which was measured for [1‐^13^C] pyruvate at 7 T,^30^ showed that the effect of a short *T*_2_* on the PSF was negligible.

While the nominal *z*‐direction resolution of the FSE sequence (1.25 mm) was double that of the DSE sequence, the true resolution was approximately four times the nominal resolution due to the PSF of the spiral acquisition design. This is because the sampling density was much lower in the corners of the 3D *k*‐space when compared with a fully sampled *k*‐space. This could be improved, as discussed in Reference 23, by adopting other 3D *k*‐space shapes. For example, simply replacing all the spirals with a 16 × 16 spiral results in a 16 × 16 × 16 cylindrical *k*‐space with 1.5 mm FWHM in the *z*‐direction PSF, which is very close to the nominal *z*‐direction resolution of 1.25 mm; however, this comes at the cost of a twofold decrease in resolution in the *xy* plane. Better spiral designs are needed to address these problems.

The key to implementation of these FSE sequences was the removal of the slice‐selection gradients from the adiabatic refocusing pulses. The phantom experiments showed that signal loss caused by residual quadratic phase variation was less than 10% for both the first and third echoes (Figure 5). However, this requires that the central frequencies of the adiabatic pulses closely match the resonance frequencies of the target metabolites in order to ensure that the majority of the resonance frequencies fall into the bottom of the phase variation well (Figure 2D). Dynamic measurements of the metabolite frequencies would be desirable, and these could be embedded between the excitation pulse and the first refocusing pulse.

A concern for these FSE sequences is that off‐resonance polarizations, for example, from untargeted metabolites or metabolites located in regions where their frequencies are off resonance, may be depleted by the transition bands of the inversion pulses. This could be avoided by using adiabatic pulses with larger bandwidths. Initial experiments using adiabatic pulses with a 1.5 kHz bandwidth were much less successful than those using pulses with a 10 kHz bandwidth. The lifetime of the observable signal with a 1.5 kHz bandwidth pulse was only half of that with 10 kHz pulses. Another potential problem is the fringe‐field effect,^18^, ^31^ where the *B*_1_ field drops rapidly at the ends of the transmit coil and results in imperfect adiabatic pulses that quickly deplete spin polarization in these areas. This phenomenon was observed in the experiments performed in vivo (Figure 8), where signals acquired using the FSE‐I sequence persisted for much longer (60 s as compared with 30 s) than those acquired using the FSE‐II sequence, which used double the number of inversion pulses. In contrast, in the phantom experiment, where the hyperpolarized [1‐^13^C] pyruvate remained at the iso‐center, both sequences resulted in signals that decayed at the same rate (supplementary Figure S2B). The fringe‐field effect could again be relieved by using pulses with a wider bandwidth, which would result in greater tolerance to *B*_1_ field inhomogeneity. A representative *B*_1_ map acquired using a Bloch‐Siegert sequence,^32^ with the same coil setup as used for the phantom and in vivo experiments, is shown in Supplementary Figure S4. A larger bandwidth requires increased RF power, and while this was not a problem in our preclinical system, where the *B*_1_ field can go beyond 500 μT, on a clinical system pulse bandwidth may be limited by the available *B*_1_ field.

Spiral‐based trajectories are relatively insensitive to motion artifacts because of rapid traversal of *k*‐space and dense sampling of the center, but are prone to off‐resonance effects. These can arise from *B*_0_ field inhomogeneity and eddy currents and usually lead to blurred and rotated images as a result of phase error accumulation during a single spiral.^33^ Moreover, with blipped *z* gradients, phase error accumulations within each stack of spirals can lead to gross image shifts and distortions. Reference scans,^34^ where the *z*‐encoding gradients were turned off, were used to correct phase errors between spirals in the acquisition train, while the phase error accumulation within each spiral was ignored as all the spirals were very short in duration. Calculations, similar to those used previously,^23^ showed that the sequences described here are immune to *B*_0_ field inhomogeneity within $\pm \frac{\pi /2}{3.12\;\mathrm{ms}}=\pm 80.12$ Hz (3.12 ms is the duration of Spirals 5 and 12 in Figure 1A, which contribute most to the phase error accumulated at the center of *k*‐space). We can usually achieve a water proton linewidth in vivo of 40–70 Hz when a spectrum is acquired from the imaging volume; therefore, ^13^C off‐resonance frequencies should be much less than ±80 Hz. In addition, eddy current effects could be measured separately and removed retrospectively in the reconstruction process.^35^

The lower SNR for the FSE‐I pulse sequence, when compared with the DSE sequence, reflects its better resolution (Figure 4B). Theoretically the DSE sequence should give an SNR that is $\sqrt{2}$ times that of the FSE‐I SNR, if signal loss due to *T*_2_‐ and *T*_2_*‐dependent relaxation is neglected and rectangular slice profiles are assumed. However, the DSE SNR for the central slices was only about 1.2 times that of FSE‐I sequence. This is because the signals are also weighted by the PSFs of the two sequences. The DSE/FSE‐I signal ratio for the central slice, calculated from the areas under the simulated PSFs, was about 1.7, which is less than the value of 2 expected from the lower nominal spatial resolution. Since twice the number of echoes were acquired in the FSE‐I sequence and $\sqrt{2}$ times the noise, the DSE/FSE‐I SNR ratio should be $\frac{1.7}{2/\sqrt{2}}\approx 1.2$, in good agreement with what we observed experimentally. Other factors can also contribute to the SNR differences, including the non‐linear phase in the FSE‐I sequence and imperfect slice selection in the DSE sequence. The FSE‐II sequence can be used to further improve the SNR of the FSE‐I sequence, although there may be faster polarization decay as the FSE‐II sequence uses more pulses. This need not be a problem for a kinetic analysis if the extra polarization loss due to imperfect refocusing pulses is incorporated into a general RF depletion term in the modified Bloch equations^36^ (e.g., take all the RF depletion caused by an excitation pulse and the following train of inversion pulses as a single term). The FSE‐II sequence improved the SNR substantially in phantom experiments and in two out of the three experiments performed in vivo with hyperpolarized [1‐^13^C] pyruvate. The reason that we failed to see an SNR improvement in the third mouse was not clear, but we infer that in this animal the *T*_2_ of the [1‐^13^C] lactate resonance was shorter. Faster‐decaying signals during the echo train may eliminate the benefit of acquiring signal from later echoes in the FSE‐II sequence, where the first echo in the repeated echo train appears 152.84 ms after the excitation pulse. Assuming an echo train with perfectly refocused spin echoes, the *T*_2_ will need to be at least 140 ms for there to be a gain in SNR by averaging signal from the later echoes. We have measured a *T*_2_ for [1‐^13^C] lactate in this tumor model of 170 ms.^37^

These pulse sequences have the potential for clinical translation, where they would benefit from the longer *T*_2_ times at lower fields. Specific absorption rate (SAR) is a potential problem; however, the pulse bandwidth could be reduced and the adiabatic refocusing pulses could be replaced with non‐selective composite pulses,^38^ although obtaining phase uniformity and refocusing performance in inhomogeneous *B*_0_ fields would be challenging.^39^ The number of pulses in these sequences is already much smaller when compared with clinical FSE sequences, such as single‐shot FSE and CUBE.^40^, ^41^ The repetition rate could also be reduced; for example, in a clinical study, where 2D imaging was used, the TR was 5 s.^4^ Clinical scanners are usually limited to much lower gradient strengths and slew rates than those used here, which may lead to long spiral durations and hence unacceptable image distortion in the *z* direction. However, translation could still be possible if the FOV were kept small to cover a restricted region of interest so that short spirals could be used. Fringe‐field effects, where the subject is not fully covered by the transmission coil, could be more of a problem. Spin polarization in a labeled molecule could be depleted rapidly if moving through a region with a rapidly varying *B*_1_ field. Potential solutions to this problem include better‐designed ^13^C transmission coils that achieve a well‐defined profile, such that the transition region, where the *B*_1_ field drops off rapidly, is limited, and multichannel transmission to achieve more uniform in‐plane polarization inversion.

## 5 CONCLUSIONS

We have described a single‐shot 3D FSE pulse sequence, which made more efficient use of the spin polarization and doubled the nominal *z*‐direction resolution when compared with a similar sequence employing a DSE design. An alternative sequence that regained the SNR lost due to the improved spatial resolution has been described. Both sequences depend on the use of echoes formed by unpaired adiabatic refocusing pulses, where the quadratic phase twist is minimized by removing slice‐selection gradients from the refocusing pulses. Although the primary applications of these sequences will be in pre‐clinical studies, unpaired adiabatic pulses have the potential to be used for ^13^C MRSI measurements in the clinic.

## Supporting information


**Supporting Figure S1:** FSE‐II pulse sequence. This is an extension of the FSE‐I sequence shown in Figure 1A. Signal is acquired from four additional stacks of spiral acquisitions. These 4 extra stacks have the same phase encodings in the z direction as in the first 4 stacks and the signals are then averaged in order to improve SNR. The FSE‐II sequence hence shares the same k‐space trajectory as the FSE‐I pulse sequence, which is shown in Figure 1B.
**Supporting Figure S2:** Adiabatic pulse calibration. (A) A pulse‐acquire sequence was used to calibrate the adiabatic pulse on a phantom filled with 5 M thermally polarized [1‐^13^C]lactate. The pulse was applied over a range of frequency offsets (0 to 4500 Hz) with varying B_1_ field strengths (40 to 470 μT). Higher signal indicates worse inversion performance of the pulse. The signals were normalized to the maximum. (B) The FSE‐I and II pulse sequences, which use 4 and 8 refocusing pulses respectively, were used to acquire signal from a phantom injected with hyperpolarized [1‐^13^C] pyruvate, where the pulses were set at the [1‐^13^C] pyruvate and [1‐^13^C] lactate resonance frequencies in alternate acquisitions, which were 1 s apart. The gradients were turned off except for the slice‐selection gradient accompanying the excitation pulse.
**Supporting Figure S3:** Comparison of the PSFs of the proposed FSE sequences and a dual spin echo (DSE) sequence described previously. (A) PSFs in XY, XZ, and YZ planes. (B) PSF in the z direction.
**Supporting Figure S4:** B_1_ map acquired at the iso‐center in the z direction from a 20 mm slice. The same coil setup was used to acquire the B_1_ map as used in the phantom and in vivo experiments. A sphere phantom (17.2 mm inner diameter) filled with thermally polarized 2 M [1‐^13^C] lactate was used. To cover a larger region in y direction, two B_1_ maps were acquired when the phantom was positioned in two different locations along the y axis (−2.5 mm and 7.5 mm) and then combined into the displayed one. A RF power of 0.25 W was used for the Bloch‐Siegert pulse.
**Supporting Figure S5:** [1‐^13^C] pyruvate images acquired in vivo in the same experiment as shown in Figure 6. The images are from a single frame (9 s from the start of acquisition). The slices are indexed in the tail to head direction.
**Supporting Figure S6:** Pyruvate and lactate signal intensities in images acquired with the FSE‐I (2 mice) and FSE‐II (3 mice) pulse sequences from tumor‐bearing mice injected with hyperpolarized [1‐^13^C]pyruvate. Signal intensities in the whole image and from the tumor region are plotted as a function of time after pyruvate injection. The mice are listed in the order of decreasing SNR in the images acquired using the FSE‐II sequence.
