# Supplementary material for: Hyperpolarized 13C spectroscopic imaging using single‐shot 3D sequences with unpaired adiabatic refocusing pulses
Source: NMR Biomed. 2018 Sep 10;31(11):e4004. doi: 10.1002/nbm.4004 (PMC6220795; doi:10.1002/nbm.4004)
Supplement: Supplementary file 1 — Supporting Figure S1: FSE‐II pulse sequence. This is an extension of the FSE‐I sequence shown in Figure 1A. Signal is acquired from four additional stacks of spiral acquisitions. These 4 extra stacks have the same phase encodings in the z direction as in the first 4 stacks and the signals are then averaged in order to improve SNR. The FSE‐II sequence hence shares the same k‐space trajectory as the FSE‐I pulse sequence, which is shown in Figure 1B. Supporting Figure S2: Adiabatic pulse calibration. (A) A pulse‐acquire sequence was used to calibrate the adiabatic pulse on a phantom filled with 5 M thermally polarized [1‐13C]lactate. The pulse was applied over a range of frequency offsets (0 to 4500 Hz) with varying B1 field strengths (40 to 470 μT). Higher signal indicates worse inversion performance of the pulse. The signals were normalized to the maximum. (B) The FSE‐I and II pulse sequences, which use 4 and 8 refocusing pulses respectively, were used to acquire signal from a phantom injected with hyperpolarized [1‐13C] pyruvate, where the pulses were set at the [1‐13C] pyruvate and [1‐13C] lactate resonance frequencies in alternate acquisitions, which were 1 s apart. The gradients were turned off except for the slice‐selection gradient accompanying the excitation pulse. Supporting Figure S3: Comparison of the PSFs of the proposed FSE sequences and a dual spin echo (DSE) sequence described previously. (A) PSFs in XY, XZ, and YZ planes. (B) PSF in the z direction. Supporting Figure S4: B1 map acquired at the iso‐center in the z direction from a 20 mm slice. The same coil setup was used to acquire the B1 map as used in the phantom and in vivo experiments. A sphere phantom (17.2 mm inner diameter) filled with thermally polarized 2 M [1‐13C] lactate was used. To cover a larger region in y direction, two B1 maps were acquired when the phantom was positioned in two different locations along the y axis (−2.5 mm and 7.5 mm) and then combined into the displayed one. A RF pow [file NBM-31-na-s001.docx]

**Supporting Information**

**High resolution hyperpolarized ^13^C spectroscopic imaging using**

**optimized single shot 3D sequences**

Jiazheng Wang^1^, Richard L. Hesketh^1^, Alan J. Wright^1^, and Kevin M. Brindle^1,2^

**Supplementary Figures:**

**
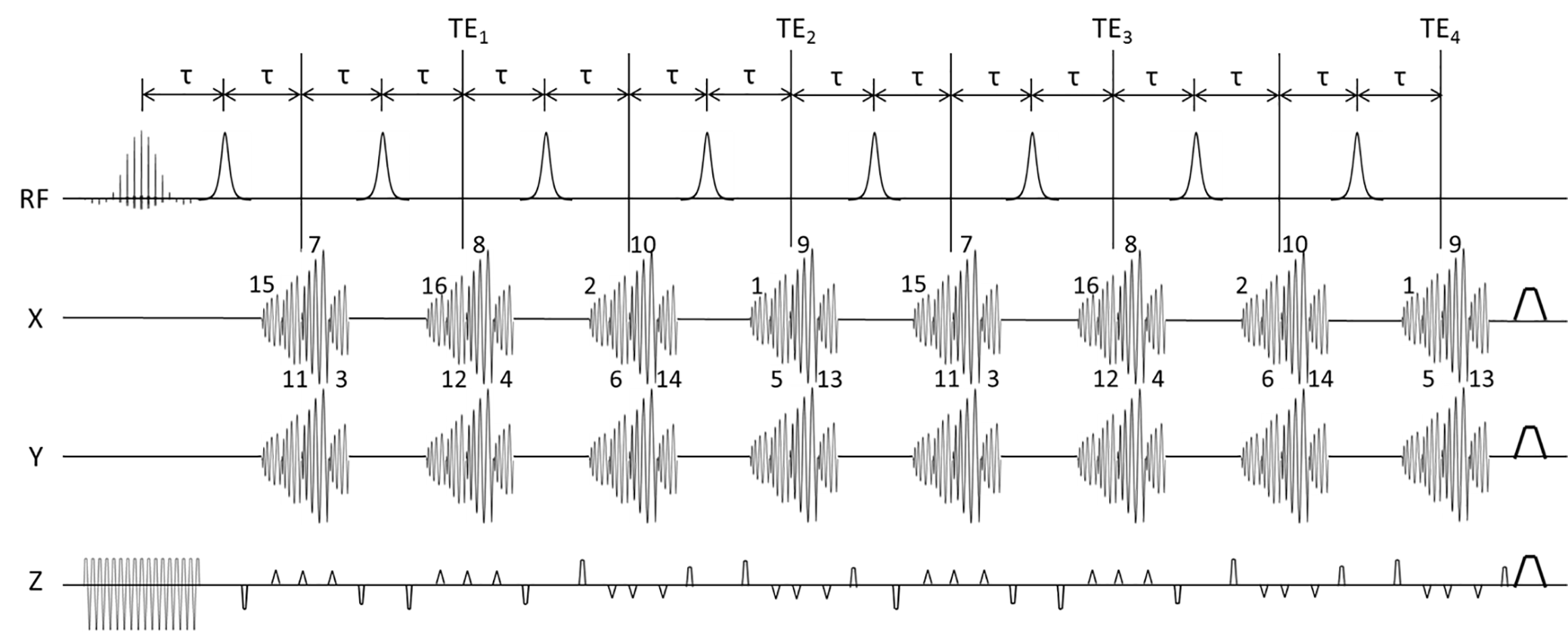
**

**Supporting Figure S1:** FSE-II pulse sequence. This is an extension of the FSE-I sequence shown in Figure 1A. Signal is acquired from four additional stacks of spiral acquisitions. These 4 extra stacks have the same phase encoding in the z direction as in the first 4 stacks and the signals are then averaged in order to improve SNR. The FSE-II sequence hence shares the same k-space trajectory as the FSE-I pulse sequence, which is shown in Figure 1B.


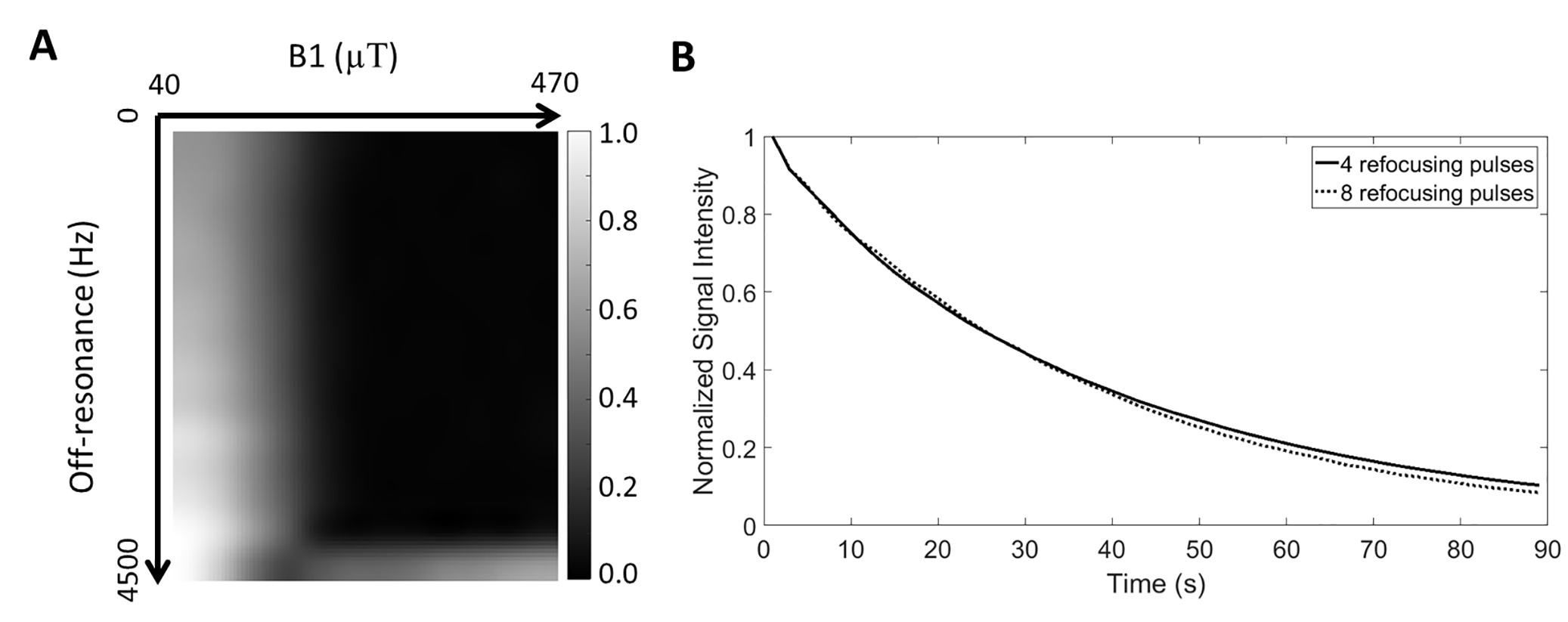


**Supporting Figure S2:** Adiabatic pulse calibration. (A) A pulse-acquire sequence was used to calibrate the adiabatic pulse on a phantom filled with 5 M thermally polarized [1-^13^C]lactate. The pulse was applied over a range of frequency offsets (0 to 4500 Hz) with varying B_1_ field strengths (40 to 470 µT). Higher signal indicates worse inversion performance of the pulse. The signals were normalized to the maximum. (B) The FSE-I and II pulse sequences, which use 4 and 8 refocusing pulses respectively, were used to acquire signal from a phantom injected with hyperpolarized [1-^13^C]pyruvate, where the pulses were set at the [1-^13^C]pyruvate and [1-^13^C]lactate resonance frequencies in alternate acquisitions, which were 1 s apart. The gradients were turned off except for the slice-selection gradient accompanying the excitation pulse.


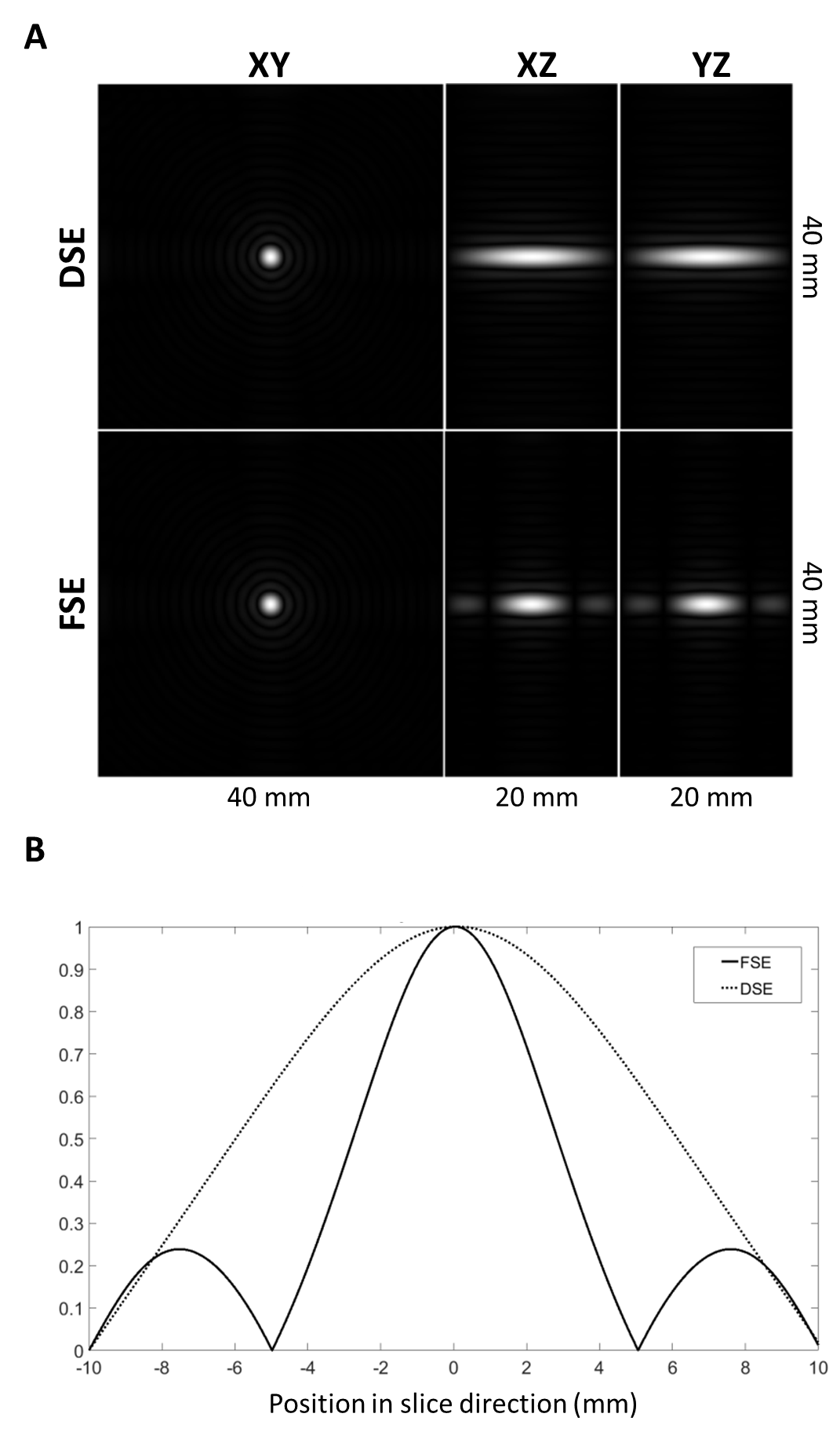


**Supporting Figure S3:** Comparison of the simulated PSFs of the proposed FSE sequences and a dual spin echo (DSE) sequence described previously. (A) PSFs in XY, XZ, and YZ planes. (B) PSF in the z direction.


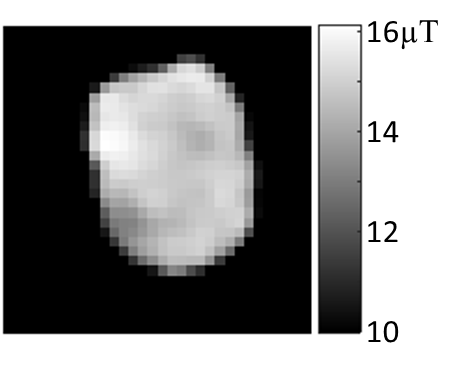


**Supporting Figure S4:** B_1_ map acquired at the iso-center in the z direction from a 20 mm slice through a sphere phantom (17.2 mm inner diameter) filled with thermally polarized 2 M [1-^13^C]lactate. The same coil setup was used to acquire the B_1_ map as was used in the phantom and in vivo experiments. To cover a larger region in the y direction, two B_1_ maps were acquired when the phantom was positioned in two different locations along the y axis (-2.5 mm and 7.5 mm) and then combined to give the displayed image. A RF power of 0.25 W was used for the Bloch-Siegert pulse.


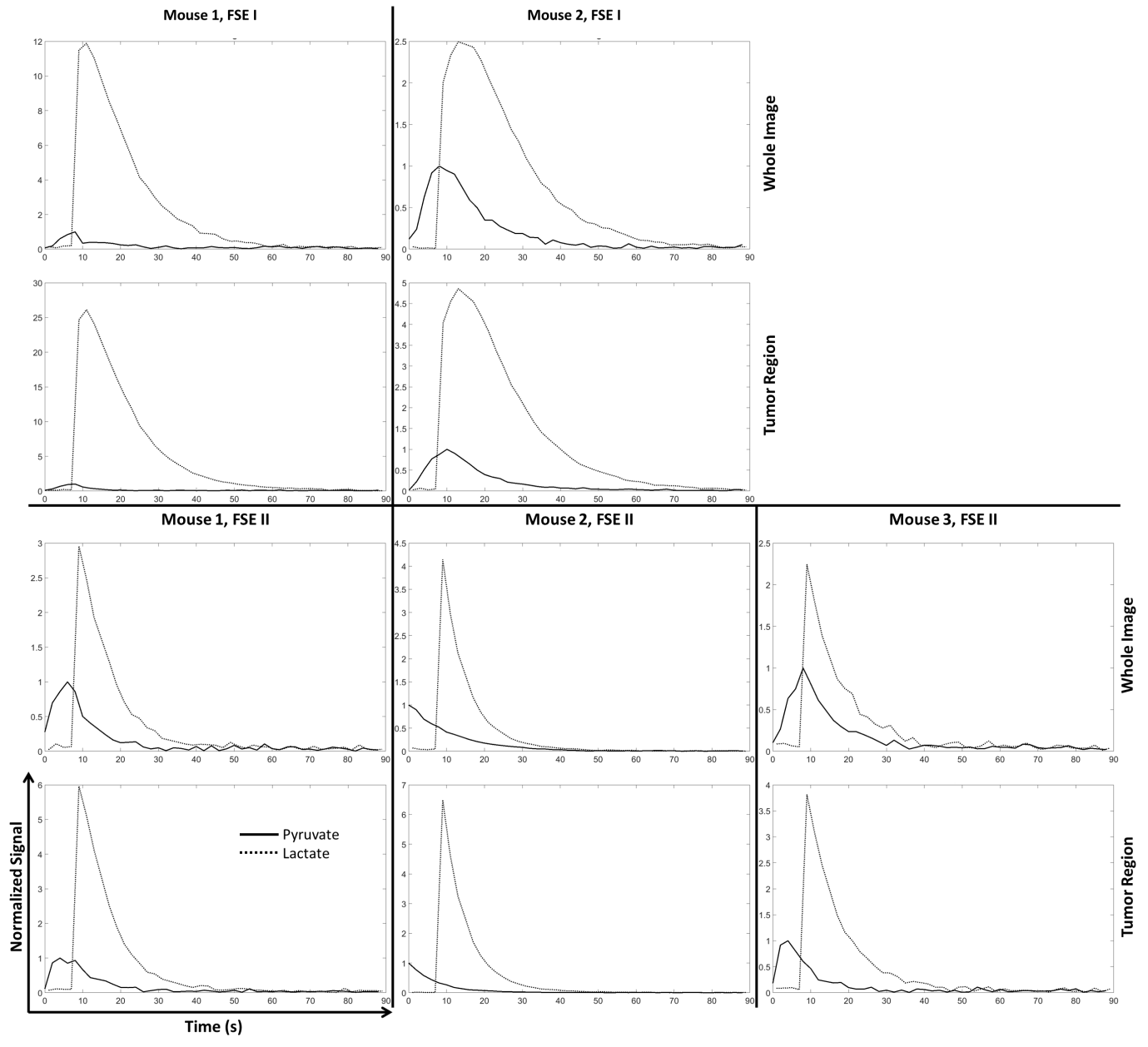


**Supporting Figure S5:** Pyruvate and lactate signal intensities in images acquired with the FSE-I (2 mice) and FSE-II (3 mice) pulse sequences from tumor-bearing mice injected with hyperpolarized [1-^13^C]pyruvate. Signal intensities in the whole image and from the tumor region are plotted as a function of time after pyruvate injection. The mice are listed in the order of decreasing SNR in the images acquired using the FSE-II sequence.


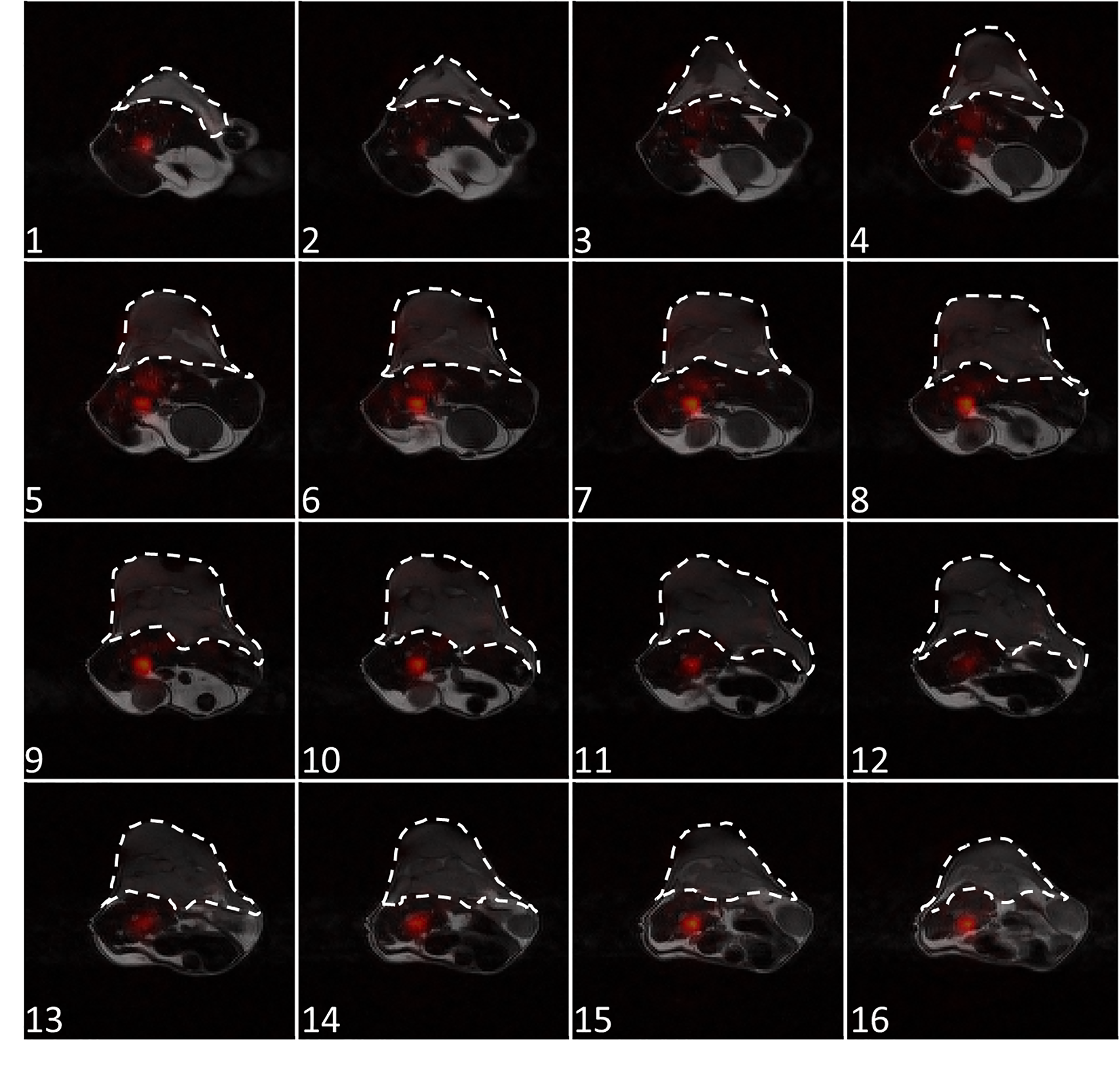


**Supporting Figure S6:** [1-^13^C]pyruvate images acquired in vivo in the same experiment as shown in Figure 6. The images are from a single frame (9 s from the start of acquisition). The slices are indexed in the tail to head direction.
